# Tris(4-*tert*-butyl­phen­yl)phosphine oxide

**DOI:** 10.1107/S1600536809052155

**Published:** 2009-12-19

**Authors:** Yin-Ge Hao, Jin-Cai Yao, Jun-Xian Li, Yu-Xin He, Yu-Qing Zhang

**Affiliations:** a Key Laboratory of Polymer Science and Nanotechnology, Henan University of Science and Technology, Luoyang 471003, People’s Republic of China; b College of Chemistry and Chemical Engineering, Luoyang Normal University, Luoyang 471022, People’s Republic of China

## Abstract

In the title compound, C_30_H_39_OP, the P=O bond length is 1.4866 (12) Å and the P—C bond lengths range from 1.804 (2) to 1.808 (13) Å. The molecle is located on a crystallographic mirror plane. The methyl groups of one *tert*-butyl group are disordered over two sites in a 0.776 (4):0.224 (4) ratio.

## Related literature

For applications of phosphine ligands in palladium-catalysed syntheses, see: Buchwald *et al.* (2006 ▶); Surry & Buchwald (2008 ▶); Xu *et al.* (2009 ▶). For related structures, see: Baures & Silverton (1990 ▶); Shawkataly *et al.* (2009 ▶). For the synthesis, see: Issleib & Brack (1954 ▶).
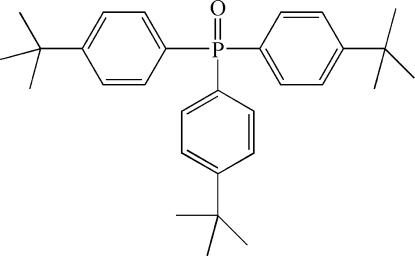


## Experimental

### 

#### Crystal data

C_30_H_39_OP
*M* *_r_* = 446.58
Orthorhombic, 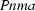

*a* = 11.7986 (10) Å
*b* = 20.9246 (18) Å
*c* = 10.5204 (9) Å
*V* = 2597.3 (4) Å^3^
*Z* = 4
Mo *K*α radiation
μ = 0.13 mm^−1^
*T* = 294 K
0.45 × 0.43 × 0.42 mm

#### Data collection

Bruker SMART APEX CCD area-detector diffractometer
Absorption correction: multi-scan (*SADABS*; Sheldrick, 1996 ▶) *T*_min_ = 0.946, *T*_max_ = 0.949
17327 measured reflections
2485 independent reflections
2143 reflections with *I* > 2σ(*I*)
*R*_int_ = 0.027

#### Refinement

*R*[*F* ^2^ > 2σ(*F* ^2^)] = 0.042
*wR*(*F* ^2^) = 0.117
*S* = 1.03
2485 reflections
161 parameters
18 restraints
H-atom parameters constrained
Δρ_max_ = 0.38 e Å^−3^
Δρ_min_ = −0.30 e Å^−3^

### 

Data collection: *SMART* (Bruker, 2004 ▶); cell refinement: *SAINT* (Bruker, 2004 ▶); data reduction: *SAINT*; program(s) used to solve structure: *SHELXS97* (Sheldrick, 2008 ▶); program(s) used to refine structure: *SHELXL97* (Sheldrick, 2008 ▶); molecular graphics: *SHELXTL* (Sheldrick, 2008 ▶); software used to prepare material for publication: *SHELXTL* and *PLATON* (Spek, 2009 ▶).

## Supplementary Material

Crystal structure: contains datablocks global, I. DOI: 10.1107/S1600536809052155/si2225sup1.cif

Structure factors: contains datablocks I. DOI: 10.1107/S1600536809052155/si2225Isup2.hkl

Additional supplementary materials:  crystallographic information; 3D view; checkCIF report

## Figures and Tables

**Fig. 1.**
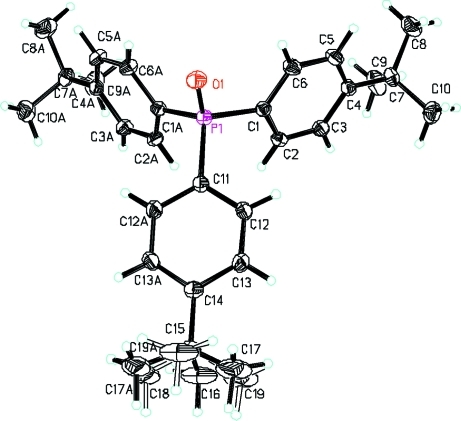
The molecular structure of the title compound with displacement ellipsoids at the 30% probability level (Symmetry code A: x, -y + 1/2, z).

## supplementary crystallographic information

### Comment

Arylphosphines are the most frequently used as ligands in transition metal catalysis (Buchwald *et al.*, 2006; Surry & Buchwald 2008; Xu *et al.*, 2009). While preparing tris(4-*tert*-butylphenyl) phosphines, we have obtained the title compound as a side product.

The title compound, C_30_H_39_OP, has a P=O bond length of 1.4866 (12) Å. The P—C bond lengths range from 1.804 (2) to 1.808 (13) Å. It is located on a crystallographic mirror plane. All the bond distances and angles in the structure are within normal ranges, similar to those found in the related compounds (Baures & Silverton 1990; Shawkataly *et al.*, 2009). The methyl groups of one *tert*-butyl group are disordered over two sites in a 0.776 (4):0.224 (4) ratio.

### Experimental

The title compound was obtained as a side product from the reaction of PCl_3_ and 4-C(CH_3_)_3_-C_6_H_4_—MgBr as described in the literature (Issleib & Brack 1954) and recrystallized from ethanol at room temperature to give the desired crystals suitable for single-crystal X-ray diffraction.

### Refinement

The methyl groups of one *tert*-butyl group are disordered over two sites, occupancies were refined and converged to 0.776 (4):0.224 (4). The rigid-group mode was used in refinement for the disordered components, and atomic displacement parameters were constrained for disordered components. H atoms attached to C atoms of the title compound were placed in geometrically idealized positions and treated as riding with C—H distances constrained to 0.93–0.96 Å, and with *U*_iso_(H)=1.2–1.5*U*_eq_(C).

### Crystal data

**Table:**

C_30_H_39_OP	*D*_x_ = 1.142 Mg m^−^^3^
*M**_r_* = 446.58	Mo *K*α radiation, λ = 0.71073 Å
Orthorhombic, *P**n**m**a*	Cell parameters from 5668 reflections
*a* = 11.7986 (10) Å	θ = 2.6–28.1°
*b* = 20.9246 (18) Å	µ = 0.13 mm^−^^1^
*c* = 10.5204 (9) Å	*T* = 294 K
*V* = 2597.3 (4) Å^3^	Block, colourless
*Z* = 4	0.45 × 0.43 × 0.42 mm
*F*(000) = 968	

### Data collection

**Table:**

Bruker SMART APEX CCD area-detector diffractometer	2485 independent reflections
Radiation source: fine-focus sealed tube	2143 reflections with *I* > 2σ(*I*)
graphite	*R*_int_ = 0.027
phi and ω scans	θ_max_ = 25.5°, θ_min_ = 2.6°
Absorption correction: multi-scan (*SADABS*; Sheldrick, 1996)	*h* = −14→14
*T*_min_ = 0.946, *T*_max_ = 0.949	*k* = −25→25
17327 measured reflections	*l* = −12→12

### Refinement

**Table:**

Refinement on *F*^2^	Primary atom site location: structure-invariant direct methods
Least-squares matrix: full	Secondary atom site location: difference Fourier map
*R*[*F*^2^ > 2σ(*F*^2^)] = 0.042	Hydrogen site location: inferred from neighbouring sites
*wR*(*F*^2^) = 0.117	H-atom parameters constrained
*S* = 1.03	*w* = 1/[σ^2^(*F*_o_^2^) + (0.0575*P*)^2^ + 1.3437*P*] where *P* = (*F*_o_^2^ + 2*F*_c_^2^)/3
2485 reflections	(Δ/σ)_max_ < 0.001
161 parameters	Δρ_max_ = 0.38 e Å^−^^3^
18 restraints	Δρ_min_ = −0.30 e Å^−^^3^

### Special details

**Table:**

Geometry. All e.s.d.'s (except the e.s.d. in the dihedral angle between two l.s. planes)are estimated using the full covariance matrix. The cell e.s.d.'s are takeninto account individually in the estimation of e.s.d.'s in distances, anglesand torsion angles; correlations between e.s.d.'s in cell parameters are onlyused when they are defined by crystal symmetry. An approximate (isotropic)treatment of cell e.s.d.'s is used for estimating e.s.d.'s involving l.s. planes.
Refinement. Refinement of *F*^2^ against ALL reflections. The weighted *R*-factor *wR* and goodness of fit *S* are based on *F*^2^, conventional *R*-factors *R* are based on *F*, with *F* set to zero for negative *F*^2^. The threshold expression of *F*^2^ > σ(*F*^2^) is used only for calculating *R*-factors(gt) *etc*. and is not relevant to the choice of reflections for refinement. *R*-factors based on *F*^2^ are statistically about twice as large as those based on *F*, and *R*-factors based on ALL data will be even larger.

### Fractional atomic coordinates and isotropic or equivalent isotropic displacement parameters (Å^2^)

**Table:**

	*x*	*y*	*z*	*U*_iso_*/*U*_eq_	Occ. (<1)
C1	0.77025 (14)	0.31883 (8)	0.30317 (15)	0.0298 (4)	
C2	0.66436 (14)	0.33374 (8)	0.35431 (16)	0.0340 (4)	
H2	0.6014	0.3093	0.3328	0.041*	
C3	0.65284 (15)	0.38473 (8)	0.43698 (17)	0.0370 (4)	
H3	0.5814	0.3944	0.4689	0.044*	
C4	0.74507 (15)	0.42213 (8)	0.47393 (16)	0.0342 (4)	
C5	0.84902 (15)	0.40734 (9)	0.42064 (17)	0.0400 (4)	
H5	0.9119	0.4319	0.4419	0.048*	
C6	0.86199 (14)	0.35678 (9)	0.33627 (17)	0.0381 (4)	
H6	0.9329	0.3483	0.3016	0.046*	
C7	0.72901 (17)	0.47686 (9)	0.56942 (18)	0.0422 (4)	
C8	0.8417 (2)	0.50323 (12)	0.6179 (2)	0.0656 (7)	
H8A	0.8839	0.4696	0.6579	0.098*	
H8B	0.8276	0.5366	0.6784	0.098*	
H8C	0.8844	0.5201	0.5478	0.098*	
C9	0.6599 (2)	0.45282 (11)	0.6836 (2)	0.0654 (7)	
H9A	0.5866	0.4390	0.6553	0.098*	
H9B	0.6513	0.4868	0.7443	0.098*	
H9C	0.6988	0.4176	0.7228	0.098*	
C10	0.6641 (2)	0.53167 (10)	0.5049 (2)	0.0592 (6)	
H10A	0.7100	0.5498	0.4387	0.089*	
H10B	0.6467	0.5640	0.5667	0.089*	
H10C	0.5950	0.5155	0.4691	0.089*	
C11	0.6881 (2)	0.2500	0.0829 (2)	0.0299 (5)	
C12	0.64869 (17)	0.30649 (9)	0.03060 (18)	0.0432 (5)	
H12	0.6743	0.3453	0.0627	0.052*	
C13	0.57171 (17)	0.30618 (9)	−0.06884 (18)	0.0453 (5)	
H13	0.5472	0.3450	−0.1021	0.054*	
C14	0.5301 (2)	0.2500	−0.1204 (2)	0.0342 (5)	
C15	0.4417 (2)	0.2500	−0.2271 (2)	0.0433 (6)	
C16	0.3243 (4)	0.2500	−0.1637 (5)	0.0934 (17)	0.776 (4)
H16A	0.3112	0.2906	−0.1240	0.140*	0.776 (4)
H16B	0.2671	0.2500	−0.2269	0.140*	0.776 (4)
C17	0.4487 (4)	0.3089 (2)	−0.3094 (4)	0.1069 (16)	0.776 (4)
H17A	0.3956	0.3054	−0.3782	0.160*	0.776 (4)
H17B	0.5240	0.3129	−0.3429	0.160*	0.776 (4)
H17C	0.4309	0.3460	−0.2594	0.160*	0.776 (4)
C18	0.5182 (14)	0.2500	−0.3521 (17)	0.0934 (17)	0.224 (4)
H18A	0.4698	0.2500	−0.4229	0.140*	0.224 (4)
H18B	0.5516	0.2086	−0.3543	0.140*	0.224 (4)
C19	0.3708 (5)	0.3102 (7)	−0.2313 (5)	0.1069 (16)	0.224 (4)
H19A	0.3178	0.3074	−0.3003	0.160*	0.224 (4)
H19B	0.4193	0.3465	−0.2436	0.160*	0.224 (4)
H19C	0.3303	0.3148	−0.1526	0.160*	0.224 (4)
O1	0.91419 (9)	0.2500 (7)	0.15255 (12)	0.0397 (4)	
P1	0.79669 (5)	0.2500 (7)	0.20357 (5)	0.02885 (19)	

### Atomic displacement parameters (Å^2^)

**Table:**

	*U*^11^	*U*^22^	*U*^33^	*U*^12^	*U*^13^	*U*^23^
C1	0.0308 (8)	0.0304 (8)	0.0281 (8)	−0.0013 (7)	−0.0028 (6)	0.0019 (7)
C2	0.0283 (8)	0.0345 (9)	0.0393 (9)	−0.0042 (7)	−0.0036 (7)	−0.0025 (7)
C3	0.0323 (9)	0.0385 (9)	0.0401 (9)	0.0005 (7)	0.0026 (7)	−0.0037 (8)
C4	0.0393 (9)	0.0304 (9)	0.0327 (9)	−0.0023 (7)	−0.0031 (7)	0.0006 (7)
C5	0.0341 (9)	0.0405 (10)	0.0455 (10)	−0.0099 (8)	−0.0034 (8)	−0.0057 (8)
C6	0.0299 (9)	0.0429 (10)	0.0414 (9)	−0.0036 (7)	0.0022 (7)	−0.0047 (8)
C7	0.0487 (11)	0.0362 (9)	0.0418 (10)	−0.0038 (8)	0.0006 (8)	−0.0080 (8)
C8	0.0646 (15)	0.0595 (14)	0.0728 (15)	−0.0078 (12)	−0.0120 (12)	−0.0304 (12)
C9	0.0945 (19)	0.0592 (14)	0.0425 (11)	−0.0148 (13)	0.0137 (12)	−0.0147 (10)
C10	0.0685 (14)	0.0424 (12)	0.0665 (14)	0.0090 (10)	0.0018 (12)	−0.0086 (11)
C11	0.0328 (12)	0.0310 (12)	0.0260 (11)	0.000	0.0004 (9)	0.000
C12	0.0580 (12)	0.0298 (9)	0.0418 (10)	−0.0022 (8)	−0.0149 (9)	−0.0015 (7)
C13	0.0597 (12)	0.0328 (10)	0.0434 (10)	0.0061 (9)	−0.0153 (9)	0.0027 (8)
C14	0.0334 (13)	0.0424 (13)	0.0270 (11)	0.000	0.0005 (10)	0.000
C15	0.0486 (16)	0.0461 (15)	0.0352 (14)	0.000	−0.0122 (12)	0.000
C16	0.055 (3)	0.157 (5)	0.068 (3)	0.000	−0.026 (2)	0.000
C17	0.121 (3)	0.117 (3)	0.083 (3)	−0.045 (3)	−0.065 (2)	0.053 (2)
C18	0.055 (3)	0.157 (5)	0.068 (3)	0.000	−0.026 (2)	0.000
C19	0.121 (3)	0.117 (3)	0.083 (3)	−0.045 (3)	−0.065 (2)	0.053 (2)
O1	0.0300 (9)	0.0473 (10)	0.0419 (10)	0.000	0.0059 (7)	0.000
P1	0.0270 (3)	0.0314 (3)	0.0282 (3)	0.000	0.0000 (2)	0.000

### Geometric parameters (Å, °)

**Table:**

C1—C6	1.387 (2)	C11—P1	1.804 (2)
C1—C2	1.396 (2)	C12—C13	1.385 (3)
C1—P1	1.808 (13)	C12—H12	0.9300
C2—C3	1.383 (2)	C13—C14	1.384 (2)
C2—H2	0.9300	C13—H13	0.9300
C3—C4	1.396 (2)	C14—C13^i^	1.384 (2)
C3—H3	0.9300	C14—C15	1.533 (3)
C4—C5	1.384 (3)	C15—C17^i^	1.509 (4)
C4—C7	1.535 (2)	C15—C17	1.509 (4)
C5—C6	1.389 (2)	C15—C19^i^	1.512 (13)
C5—H5	0.9300	C15—C19	1.512 (13)
C6—H6	0.9300	C15—C16	1.537 (6)
C7—C8	1.527 (3)	C15—C18	1.596 (19)
C7—C9	1.537 (3)	C16—H16A	0.9600
C7—C10	1.537 (3)	C16—H16B	0.9471
C8—H8A	0.9600	C17—H17A	0.9600
C8—H8B	0.9600	C17—H17B	0.9600
C8—H8C	0.9600	C17—H17C	0.9600
C9—H9A	0.9600	C18—H18A	0.9378
C9—H9B	0.9600	C18—H18B	0.9517
C9—H9C	0.9600	C19—H19A	0.9600
C10—H10A	0.9600	C19—H19B	0.9600
C10—H10B	0.9600	C19—H19C	0.9600
C10—H10C	0.9600	O1—P1	1.4866 (12)
C11—C12^i^	1.384 (2)	P1—C1^i^	1.808 (13)
C11—C12	1.384 (2)		
			
C6—C1—C2	118.28 (15)	C14—C13—H13	118.9
C6—C1—P1	117.83 (17)	C12—C13—H13	118.9
C2—C1—P1	123.78 (18)	C13—C14—C13^i^	116.2 (2)
C3—C2—C1	120.19 (15)	C13—C14—C15	121.88 (11)
C3—C2—H2	119.9	C13^i^—C14—C15	121.88 (11)
C1—C2—H2	119.9	C17^i^—C15—C17	109.6 (4)
C2—C3—C4	122.08 (16)	C17^i^—C15—C19^i^	48.2 (3)
C2—C3—H3	119.0	C17—C15—C19^i^	133.9 (3)
C4—C3—H3	119.0	C17^i^—C15—C19	133.9 (3)
C5—C4—C3	116.92 (16)	C17—C15—C19	48.2 (3)
C5—C4—C7	122.78 (16)	C19^i^—C15—C19	112.7 (7)
C3—C4—C7	120.30 (16)	C17^i^—C15—C14	112.50 (18)
C4—C5—C6	121.79 (16)	C17—C15—C14	112.50 (18)
C4—C5—H5	119.1	C19^i^—C15—C14	113.5 (2)
C6—C5—H5	119.1	C19—C15—C14	113.5 (2)
C1—C6—C5	120.69 (16)	C17^i^—C15—C16	107.4 (3)
C1—C6—H6	119.7	C17—C15—C16	107.4 (3)
C5—C6—H6	119.7	C19^i^—C15—C16	60.9 (4)
C8—C7—C4	112.37 (16)	C19—C15—C16	60.9 (4)
C8—C7—C9	108.61 (18)	C14—C15—C16	107.2 (2)
C4—C7—C9	109.45 (15)	C17^i^—C15—C18	59.7 (3)
C8—C7—C10	108.16 (17)	C17—C15—C18	59.7 (3)
C4—C7—C10	109.22 (15)	C19^i^—C15—C18	106.8 (4)
C9—C7—C10	108.98 (19)	C19—C15—C18	106.8 (4)
C7—C8—H8A	109.5	C14—C15—C18	102.6 (6)
C7—C8—H8B	109.5	C16—C15—C18	150.1 (6)
H8A—C8—H8B	109.5	C15—C16—H16A	109.5
C7—C8—H8C	109.5	C15—C16—H16B	109.7
H8A—C8—H8C	109.5	H16A—C16—H16B	101.0
H8B—C8—H8C	109.5	C15—C17—H17A	109.5
C7—C9—H9A	109.5	C15—C17—H17B	109.5
C7—C9—H9B	109.5	C15—C17—H17C	109.5
H9A—C9—H9B	109.5	C15—C18—H18A	108.1
C7—C9—H9C	109.5	C15—C18—H18B	104.7
H9A—C9—H9C	109.5	H18A—C18—H18B	103.5
H9B—C9—H9C	109.5	C15—C19—H16A	90.7
C7—C10—H10A	109.5	C15—C19—H19A	109.5
C7—C10—H10B	109.5	C15—C19—H19B	109.5
H10A—C10—H10B	109.5	H19A—C19—H19B	109.5
C7—C10—H10C	109.5	C15—C19—H19C	109.5
H10A—C10—H10C	109.5	H19A—C19—H19C	109.5
H10B—C10—H10C	109.5	H19B—C19—H19C	109.5
C12^i^—C11—C12	117.3 (2)	O1—P1—C1	111.72 (19)
C12^i^—C11—P1	121.2 (5)	O1—P1—C11	114.10 (9)
C12—C11—P1	121.2 (5)	C1—P1—C11	106.6 (5)
C11—C12—C13	121.07 (17)	O1—P1—C1^i^	111.72 (16)
C11—C12—H12	119.5	C1—P1—C1^i^	105.59 (10)
C13—C12—H12	119.5	C11—P1—C1^i^	106.6 (5)
C14—C13—C12	122.14 (17)		
			
C6—C1—C2—C3	−0.8 (2)	C13—C14—C15—C17	28.3 (4)
P1—C1—C2—C3	175.4 (4)	C13^i^—C14—C15—C17	−152.7 (3)
C1—C2—C3—C4	−1.1 (3)	C13—C14—C15—C19^i^	−154.7 (4)
C2—C3—C4—C5	2.1 (3)	C13^i^—C14—C15—C19^i^	24.3 (5)
C2—C3—C4—C7	−177.92 (16)	C13—C14—C15—C19	−24.3 (5)
C3—C4—C5—C6	−1.4 (3)	C13^i^—C14—C15—C19	154.7 (4)
C7—C4—C5—C6	178.71 (16)	C13—C14—C15—C16	−89.5 (2)
C2—C1—C6—C5	1.5 (3)	C13^i^—C14—C15—C16	89.5 (2)
P1—C1—C6—C5	−174.9 (3)	C13—C14—C15—C18	90.5 (2)
C4—C5—C6—C1	−0.5 (3)	C13^i^—C14—C15—C18	−90.5 (2)
C5—C4—C7—C8	−11.6 (3)	C6—C1—P1—O1	−9.6 (3)
C3—C4—C7—C8	168.51 (18)	C2—C1—P1—O1	174.21 (16)
C5—C4—C7—C9	−132.3 (2)	C6—C1—P1—C11	−134.9 (3)
C3—C4—C7—C9	47.8 (2)	C2—C1—P1—C11	48.9 (6)
C5—C4—C7—C10	108.5 (2)	C6—C1—P1—C1^i^	112.0 (2)
C3—C4—C7—C10	−71.5 (2)	C2—C1—P1—C1^i^	−64.1 (2)
C12^i^—C11—C12—C13	0.9 (4)	C12^i^—C11—P1—O1	86.9 (3)
P1—C11—C12—C13	174.97 (17)	C12—C11—P1—O1	−86.9 (3)
C11—C12—C13—C14	0.3 (3)	C12^i^—C11—P1—C1	−149.3 (4)
C12—C13—C14—C13^i^	−1.4 (4)	C12—C11—P1—C1	36.9 (4)
C12—C13—C14—C15	177.7 (2)	C12^i^—C11—P1—C1^i^	−36.9 (4)
C13—C14—C15—C17^i^	152.7 (3)	C12—C11—P1—C1^i^	149.3 (4)
C13^i^—C14—C15—C17^i^	−28.3 (4)		

Symmetry codes: (i) *x*, −*y*+1/2, *z*.
